# Dietary Practices and Associated Factors Among Lactating Mothers in Sodo Zuriya District, Southern Ethiopia: A Community‐Based Cross‐Sectional Study

**DOI:** 10.1155/jnme/3693610

**Published:** 2026-09-16

**Authors:** Habtamu Elias Jorge, Merid Assefa Awato, Thilagavathi Thangavel

**Affiliations:** ^1^ School of Public Health, Wolaita Sodo University College of Health Science and Medicine, Wolaita Sodo, Ethiopia

**Keywords:** community-based, dietary practice, Ethiopia, factors, lactating mothers, Sodo Zuriya district

## Abstract

**Background:**

Poor dietary practices among lactating mothers can induce metabolic disturbances in infants early in life, particularly those related to nutrition, which result in irreversible physiologic changes. Although maternal nutrition is widely studied, few studies have focused on the dietary practices against the WHO/FAO recommendations for additional meal intake during lactation. This study aims to assess dietary practices and associated factors among lactating mothers based on recommended additional meal intake in the Sodo Zuriya district, South Ethiopia.

**Methods:**

A community‐based cross‐sectional study of 627 lactating mothers caring for infants under 6 months of age was conducted. Data were collected through a structured questionnaire and analyzed using SPSS v20. Binary logistic regression analyses were employed to identify factors associated with dietary practices. Adjusted odds ratios with 95% CIs at a *p* value of < 0.05 were used to declare a statistically significant association.

**Results:**

In this study, only 50.2% of the lactating mothers had good dietary practices. Counseling about nutrition (AOR = 0.28 (0.15–52)), avoiding any food group (AOR = 0.59 (0.36–0.95)), frequency of antenatal care (AOR = 0.36 (0.15–0.86)), knowledge about foods (AOR = 1.99 (1.05–3.77)) and being taken as additional (AOR = 0.17 (0.03–0.99)), milk/milk product intake per day (AOR = 0.56 (0.36‐‐0.88)), vitamin A‐rich fruit or vegetable intake per week (AOR = 2.06 (1.01–4.21)), and staple food availability/production at household (AOR = 0.50 (0.33–0.77)) were significantly associated with dietary practices.

**Conclusions:**

The dietary practices of lactating mothers in this setting were below the national reports and recommendations of the WHO and FAO. Routine health facility counseling, debunking cultural food taboos, and promoting agricultural crop diversification are essential to improving maternal dietary quality.

## 1. Introduction

Lactation is the process of milk secretion and is often referred to as the physiological completion of the reproductive cycle in women. The nutrient needs during breastfeeding are the sum of women’s nutrient needs and status and the amount of breast milk produced. A significant maternal energy deficit may reduce the quantity of breast milk produced, but it does not affect its quality [1]. Lactating mothers have higher demands for energy and nutrients than both prepregnancy and pregnancy requirements because lactation is an energy expending mechanism [2].

The World Health Organization (WHO) advises that, to improve children’s growth, pregnant women in developing countries should take iron supplements and lactating mothers should take vitamin A supplements [3].

Poor dietary practice is the amount of energy and nutrients available in the food consumed by the lactating mothers is being below the national and WHO/FAO recommendation for lactating mothers per day. Poor dietary practices, which impact on the health and wellbeing of mothers and their children, especially lactating mothers in low‐income countries, are vulnerable groups [4, 5].

Maternal and child under nutrition are among the major developmental challenges of our times in Ethiopia as well as in contributing a large share to the global disease burden [6, 7]. One in every four lactating mothers in Ethiopia was undernourished [8].

Different literature inconsistently revealed that lactating mothers in Ethiopia are considered as a nutritionally high‐risky group due to different factors that affect their dietary practice; i.e., low family income, low educational level and low occupational status, culture, lack of frequent meal delivery and low family encouragement, limited accessibility of nutritional information and nutritional knowledge, infectious diseases, supplemental implementation gap, and health care, which will impact on the health and nutrition of children [9–11].

Most studies of human lactation have focused on the quality and quantity of milk produced or on the effects of human milk on infants. Fewer studies have targeted on the dietary practice of lactating mothers and maternal health related to lactation but they are also not focused on their recommended diet and nutrient meeting habit [12].

However, the previous studies conducted in the study area have not been analyzed based on the recommended additional meal intake and have not assessed their energy and nutrient intake. This study aimed to assess dietary practices and associated factors among lactating mothers in Sodo Zuriya district, Southern Ethiopia.

Understanding local dietary practices directly supports the strategic goals of the second National Nutrition Program of Ethiopia (NNPII) to end malnutrition by 2030. This study provides evidence for policymakers, public health planners, district health officials, and agricultural offices to design targeted interventions.

## 2. Methods and Materials

### 2.1. Study Area, Design, and Period

A community‐based cross‐sectional study was conducted in Sodo Zuriya district, Wolaita Zone, South Ethiopia, from January 30 to March 15, 2021.

### 2.2. Population and Eligibility Criteria

#### 2.2.1. Source Population

All lactating mothers in the Sodo Zuriya district.

#### 2.2.2. Study Population

Randomly selected lactating mothers caring for infants aged under 6 months.

### 2.3. Inclusion Criteria

1. All lactating mothers who were caring for < 6‐month‐old infants provided informed consent to participate in the study.
2. Permanent residents (those who lived in the study area for at least 6 months).
3. Lactating mothers who were not on medication that needed dietary modification.

### 2.4. Exclusion Criteria

1. Mothers who are severely ill and unable to respond.
2. Mothers who breastfed > 6‐month‐old infants.
3. Mothers with chronic illnesses, such as diabetes mellitus, hypertension, and overweight/obesity, may have dietary restrictions (there may be dietary modifications due to either the physician’s advice or to reduce their body weight and manage the disease).

### 2.5. Sample Size Determination and Sampling Procedure

The sample size was calculated by using the formula to estimate a single population proportion formula based on a 54.8% prevalence of not consuming extra meals among lowland lactating mothers in Raya district, Tigray, Ethiopia [13], and a 5% marginal error and 95% confidence level. Considering design effect 1.5 and adding 10% nonresponse rate, the final sample size was 627.

**FIGURE 1:**
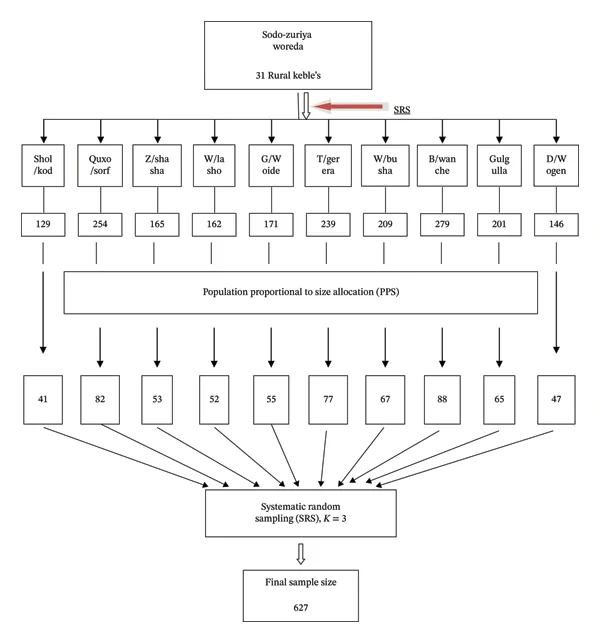
Schematic representation of the sampling technique.

A multistage random sampling technique was applied. First, out of 31 Keble’s in the district, 10 Keble’s were selected through simple random sampling. The list of all lactating mothers of all children under 2 years old was identified from the family folder in health posts in the selected kebeles, and the lactating mothers who breastfed more than 6‐month‐old infants and children were excluded from the sampling frame. Proportional allocation to population size (PPS) techniques were used for sample size allocation to the selected Keble’s. Second, a systematic sampling method was used to select lactating mothers who were included in the study from the sampling frame of each selected Kebele (Figure 1). The sampling interval *K* was identified by dividing the total number of lactating mothers who breastfed fewer than 6‐month‐old infants in the sampling frame by their allocated sample size for each selected Keble (*K* = 3). The first respondent was selected randomly from the first three listed lactating mothers, and every third case was identified in ascending order and selected until the sample size for the kebele was achieved.

### 2.6. Data Collection Methods and Tools

A structured questionnaire adapted from different studies [9, 14–16] was used for additional meal‐feeding practices, a single 24‐h recall and food frequency.

A separate questionnaire was formulated and used for weighing food records of (*n* = 30) selected lactating mothers, which were conducted for 2 days, including one weekday and one weekend day, at the mothers’ households, who volunteered [17].

The study tool or questionnaire was first prepared in English with standards by the investigator and advisors, and then translated to the Wolaitigna version by a language expert and back translated to English to maintain its consistency with another language expert.

The dietary practice assessment includes data on sociodemographic characteristics, maternal characteristics, feeding patterns of lactating mothers, food frequency, and minimum dietary diversity for women (MDD‐W)and actual dietary intake. The assessment period involves the time from when the mother gave birth until the data collection date. Ten trained health professionals collected data from randomly selected kebeles. The feeding patterns of lactating mothers were collected via a structured questionnaire. Finally, the mothers who consumed two or more additional meals during their lactation were categorized as having good dietary practices, whereas those who consumed one or no additional meals were categorized as having poor dietary practices [18]. The frequency of 9 food groups’ current consumption practices in the previous week prior to survey day recall was recorded.

A total of 10 food groups were considered in this study (staple food, legume nuts and seeds, fats and oils, milk/milk products, meat, poultry and fish, eggs, dark green leafy vegetables, vitamin A–rich fruit and vegetables, other vegetables, and other fruits) to collect 24‐h recall data from the FANTA III. The food groups consumed by the participants were recorded; participants who consumed < 5 food groups were considered not meeting the minimum diversified diet for women (MDD‐W) or low dietary diversity, whereas those who consumed ≥ 5 food groups were considered meeting the minimum diversified diet for women (MDD‐W) or high dietary diversity [19].

The mothers selected for the food weight survey were given clear information on the need for the survey, and a good rapport was created to increase the ease and comfort of the mothers, which was highly challenging. The intake of any drink or beverage or food from home for 2 days, including one weekday and one weekend day, was estimated, and all the food consumed at home was weighed and recorded by trained data collectors via an electronic kitchen scale (SF‐400) with capacity (3–7 kg) and accuracy (± 1–5 gm.) for the 30 lactating mothers randomly selected from the study sample. For each food item, raw or cooked, the total cooked weight of the household and individual portion size, and plate waste of the mother from breakfast to dinner, including all snack times, were carefully included. From the 2‐day weights of the foods consumed, the mean was calculated and analyzed as single data points on the basis of the Ethiopian Food Composition Table and SPSS version 20 [20–22]. Finally, the results were compared with the WHO‐recommended nutrient intake (RNI) for lactating women [23].

### 2.7. Data Quality Control

A 3‐day training was provided to 10 data collectors/interviewers and 3 supervisors. A pretest was conducted on 5% of the sample size in the neighboring Damot Gale District. The scales were calibrated daily.

### 2.8. Operational Definitions

Dietary practice: Eating habits (additional meal intake, dietary diversity, and energy and nutrient intake) of lactating mothers during lactation in the study setting, according to the recommendations.

Poor dietary practices: In this study, mothers did not consume additional meals or consumed only one additional meal in addition to their usual meals during their lactation [18, 24].

Good dietary practices include the following: Lactating mothers who consume at least two or more additional meals in addition to their usual meal during lactation [25].

Additional meal: Eating extra food other than regular; approximately 1.5 slices of bread extra (or 1 glass of milk and 1/2 slices of bread extra) or any food that accounts for at least 120 g of any local food per day will be considered an additional meal [26].

Lactating mother: Mother who is currently receiving breast milk for her infant aged less than 6 months.

Minimum Dietary Diversity for Women of Reproductive Age (MDD‐W): Women who consumed ≥ 5 food groups out of 10 food groups yesterday from 24 h recall data [19].

Dietary intake: the consumed food content of energy and nutrients calculated per day for lactating mothers based on the Ethiopian Food Composition Table.

### 2.9. Data Analysis

After all the relevant data were collected, the data were coded and entered into Epi Data version 3.1 software and exported to the Statistical Package for the Social Sciences (SPSS) version 20 for cleaning and further analysis. A multicollinearity test was carried out to determine the correlation between independent variables (VIF < 10 and tolerance > 0.1 were a concern). The Hosmer and Lemeshow model was used to check for model goodness of fit (closeness between the observed and model‐predicted count values, *p* = 0.441). The nutrient and energy contents of the foods consumed by the mothers were calculated on the basis of the Ethiopian Food Composition Table and analyzed via SPSS version 20 [20]. To investigate the socioeconomic, demographic, and other factors affecting the dietary practices of the mothers, multiple logistic regressions were used. Significant variables observed in the bivariate analysis (with a *p* value < 0.25) were subsequently included in the multivariable analysis. *p* values less than 0.05 along with AORs and 95% CIs were considered significant. Finally, descriptive statistics, such as frequency distributions, proportions, percentages, means, standard deviations, charts, and tables, were used to present the study results.

### 2.10. Variables

#### 2.10.1. Dependent Variable

Dietary practice.

#### 2.10.2. Independent Variables

• Socioeconomic/demographic factors: age, marital status, educational status, mother’s occupation, family size, and family income.
• Obstetric factors: antenatal care, place of delivery, postnatal care (counseling about nutrition), and family planning.
• Micronutrient supplementation: iron and vitamin A.
• Environmental sanitation: availability of latrine and source of drinking water.
• Behavioral factors: knowledge and attitudes toward nutrition.

### 2.11. Ethical Consideration

The ethical clearance was obtained from Wolaita Sodo University, School of Public Health. The official letter of support was written to the Wolaita Zone Health Office from Wolaita Sodo University, School of Public Health. Similarly, a formal letter was submitted to the Sodo Zuriya district health office from the zonal health office. Then, a letter of permission was obtained from the Sodo Zuriya District Health Office to Kebeles and Health posts. Verbal informed consent was obtained from all the participants. The rights of the participants were respected. Not being included in the study or interrupting at any time during the study was ensured. All participants were coded to ensure privacy, and their confidentiality was maintained. Participants identified during data collection as having severe dietary inadequacy, severe acute malnutrition, or severe illness due to malnutrition were provided immediate basic nutritional counseling by trained health professionals during data collection and referred to the nearby health post or health center for appropriate clinical care management and follow‐up.

## 3. Results

### 3.1. Socioeconomic Status of Lactating Mothers

A total of 606 lactating mothers participated in the study, with a response rate of 96.7%. Table 1 shows that the majority of lactating mothers in the study area (63.9%) were in the age range of 25–35 years. The mean age of the mothers was 26.5 ± 4.732 years (SD). Among the mothers, approximately 22.9% had not received formal education, 77.1% had received primary education and above, but only 1.2% had an education level of college and above. Among the households with agricultural land, approximately 94.6% had ≤ 0.5 ha of land. Approximately half of the households (49.5%) had ≤ 5 family members. With respect to livestock availability, approximately 79.2% of the respondents had different types of livestock. In terms of monthly income, the majority (82.5%) received an estimated amount of ≤ 500 ETB, and only 4% received ≥ 1500 ETB per month (Table 1).

**TABLE 1:** Socioeconomic and demographic characteristics of lactating mothers who breastfed under 6‐month‐old infants in Sodo Zuriya District, Wolaita Zone, southern Ethiopia, 2021 (*n* = 606).

Age of mothers	Frequency	Percent (%)
15–24	183	30.2
25–35	387	63.9
36–49	36	5.9
Educational status of the mother		
No formal education	139	22.9
Primary education	394	65.0
Secondary education	66	10.9
Collage and above	7	1.2
Educational status of husband/partner		
No formal education	102	16.8
Primary education	381	62.9
Secondary and above education	123	16.5
College and above	23	3.8
Estimated monthly household income (in ETB)		
≤ 500	500	82.5
501–1500	82	13.5
≥ 1500	24	4.0
Family size		
≤ 5	300	49.5
6–7	227	37.5
> 7	79	13.0
Livestock availability		
Yes	480	79.2
No	126	20.8
Occupation of the mother	Frequency	Percent
House wife	549	90.6
Farmer and daily laborer	10	1.7
Merchant	39	6.4
Government employer	8	1.3
Marital status		
Occupation of the partner/husband		
Government employ	27	4.5
Daily laborer	57	9.4
Farmer	398	65.7
Merchant	111	18.3
Other	13	2.1
Marital status		
Single	10	1.7
Married	594	98
Widowed	2	0.3

### 3.2. Obstetric Characteristics of the Respondents

Five hundred and fifty‐four (91.4%) mothers had followed antenatal care in their last pregnancy. Among the antenatal care followers, only seven (1.2%) attended once, and 269 (44.4%) mothers attended four times in their last pregnancy. Four hundred and ninety‐nine (82.3%) mothers used the modern family planning method throughout their lifetime. More than half, 369 (60.9%) of the mothers, gave birth at health facilities, 12 (1.98%), 304 (50.16%), or 53 (8.74%) at health posts, health centers, or hospitals, respectively, and the remaining 237 (39.1%) gave birth at home. Among those who gave birth at health facilities, 283 (76.7%) were counseled about nutrition before discharge from the facility, and 86 (23.3%) were not counseled about nutrition.

### 3.3. Dietary Practice of Lactating Mothers

**FIGURE 2:**
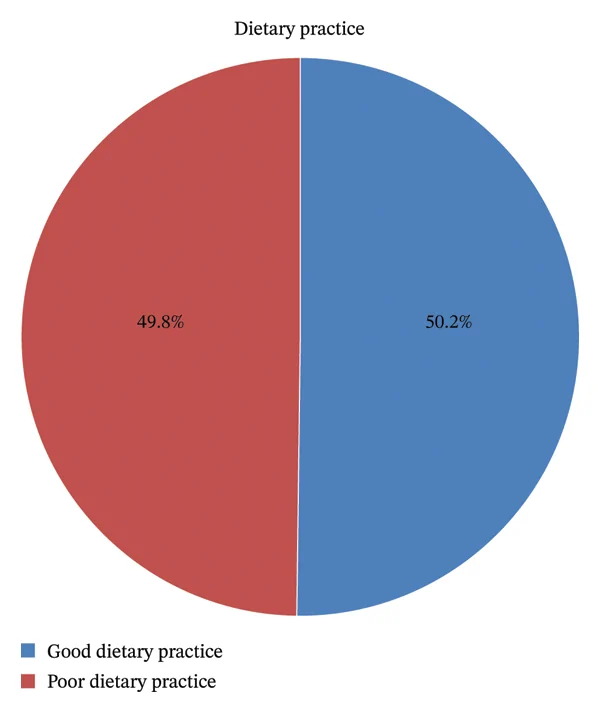
Dietary practice pattern of lactating mothers.

Among the lactating mothers, almost half (50.2%) of the lactating mothers had good dietary practices (Figure 2).

### 3.4. MDD‐W After 24 h of Dietary Recall

Only 168 (27.7%) mothers were taking a minimum of DD‐W or five or more food groups out of 10 food groups (staple foods, legumes, nuts and seeds, fats and oils, milk/milk products, meat, poultry and fish, eggs, dark green leafy vegetables, vitamin A–rich fruit and vegetables, other vegetables, and other fruits). The mean MDD‐W was 4.0 ± 1.2 SD, with a minimum of 1 and a maximum of 7 food groups (Table 2).

**TABLE 2:** Twenty‐four‐hour dietary recall data of lactating mothers (*n* = 606).

Variables		Frequency	Percent (%)
Staple food	Yes	602	99.3
	No	4	0.7

Legumes, nuts, and seeds	Yes	263	43.4
	No	343	56.6

Fats and oils	Yes	462	76.2
	No	144	23.8

Milk and milk products	Yes	403	66.5
	No	203	33.5

Meat, poultry, and fish	Yes	6	1.0
	No	600	99

Eggs	Yes	3	0.5
	No	603	99.5

Dark green leafy vegetables	Yes	228	37.6
	No	378	62.4

Vitamin A–rich fruit and vegetables	Yes	169	27.9
	No	437	72.1

Other vegetables	Yes	43	7.1
	No	563	92.9

Other fruits	Yes	116	19.1
	No	490	80.9

### 3.5. Food Frequency Per Week Before the Survey

**FIGURE 3:**
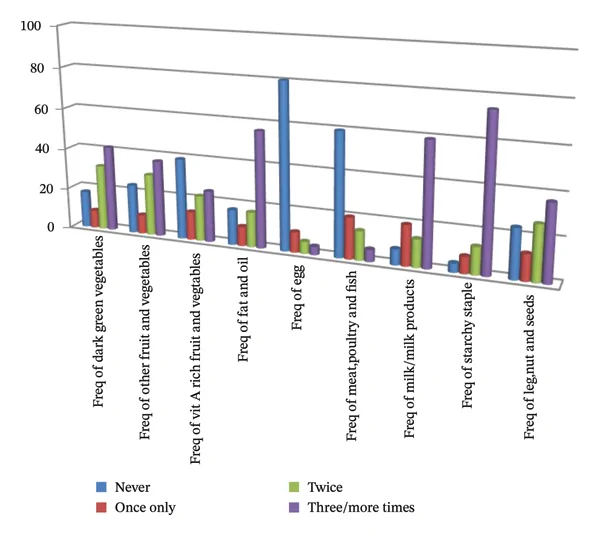
Food frequency data from the previous week to the survey date of lactating mothers (*n* = 606).

The staple foods commonly consumed in the study area were potato, sweet potato, cassava, boyina (Godere), wheat, barley, Teffi, maize, and red pea. The usual feeding habits of the mothers indicated that 3.7% of the mothers practiced fewer than 3 times per day, 4% of the mothers fed 4 times per day, and the remaining 92.4% of the mothers fed 3 times regularly. Figure 3 indicates that the majority of lactating mothers (87.6% and 92.4%) took starchy staples and milk/milk products at least once or more times in the week preceding the survey, respectively. Sixty per cent of the lactating mothers never consumed meat, poultry, or fish per week preceding the survey, and only 5.9% of the mothers consumed meat, poultry, or fish three or more times per week. Approximately 39.3% of the lactating mothers had never consumed a rich fruit or vegetable per week. The majority of the lactating mothers (82.8%) consumed fat or oil at least once or more per week (Figure 3).

### 3.6. Knowledge and Attitudes of Lactating Mothers Regarding Dietary Practices

Among the participants, 62.9% of the mothers had nutrition‐related information before and after delivery, 60.2% had information from health institutions, and the remainder had information from other sources, such as media, workplaces, and other social organizations. In terms of the type of information, 21.9% were about dietary diversity, 25.1% were about extra meal intake, and the remaining 21.9% were about the frequency of meals, animal sources of food, and fruits and vegetables.

Table 3 highlights that the majority of lactating mothers (99%, 98.5%, and 97.9%) had knowledge of the number of additional meals to be eaten, foods that can improve lactation (lactogenic), and foods to be consumed as additional foods, respectively. More than one‐fourth of lactating mothers avoid one or more food groups in their meals for traditional or cultural reasons (Table 3).

**TABLE 3:** Knowledge and attitudes of lactating mothers regarding dietary practices during lactation (*n* = 606) who breastfeed less than 6‐month‐old infants in the Sodo Zuriya district, Wolaita Zone, Ethiopia.

Variables	Frequency	Percent (%)
1. Knows which food can improve lactation		
Not known (no correct answer)	9	1.5
One correct answer	184	30.4
Two correct answers	285	47.0
Three/more correct answers	128	21.1
2. Know foods to be taken additionally.		
Not known (no correct answer)	13	2.1
One correct answer	131	21.6
Two correct answers	333	55.0
Three/more correct answers	129	21.3
3. Knows the number of additional meals that would be taken during lactation		
Not known (no correct answer)	6	1.0
One correct answer	578	95.4
Two correct answers	22	3.6
4. Do you think any food group would be avoided during lactation?		
Yes	197	32.5
No	409	67.5
Not known	—	—
5. Do you think lactating mothers could take an additional meal during their lactation?		
Yes	561	92.6
No	45	7.4
Not known	‐	‐
6. Do you think taking different types of food groups in a meal is good?		
Yes	81	13.4
No	525	86.6
Not known	‐	‐

### 3.7. Environmental Factors

Among the 606 lactating mothers, 98.3% of the households had their own latrine. A total of 84.2% had drinking water from pipe water, 12.2% were receiving drinking water from protected springs/wells, and 2.0% and 1.7% were receiving drinking water from unprotected springs/wells and rivers, respectively.

### 3.8. Micronutrient Supplementation Intake During the Last Pregnancy and After Delivery

Among all the lactating mothers in the study, nearly half (48.5%) of the mothers received vitamin A supplementation after their last delivery. The majority (84.2%) of the mothers consumed iron supplements during their last pregnancy. Among those who consumed the iron supplement, 93.1% took the iron foliate tablet for less than or equal to 3 months, and only 6.9% took it for more than 3 months (Table 4).

**TABLE 4:** Energy and other nutrient intakes of the (*n* = 30) lactating mothers.

No.	Energy and nutrients	Mean amount with SD	Maximum	Minimum	RNI for lactating mothers [23]
1	Energy (kcal)	1586.4525 ± 374.13	2227.562	1085.245	2700.69
2	Protein (g)	31.59 ± 10.62	52.373	18.01	65
3	CHO (g)	320.6 ± 86.96	465.766	197.185	290.7
4	Fat (g)	19.12 ± 11.55	47.1	6.934	69.1
5	Calcium (mg)	323 ± 126.9	517.21	137.5	1000
6	Thiamin (milligram)	1.1 ± 0.6	2.3	0.503	1.5
7	Iron (milligram)	25.08 ± 17.69	59.422	12.144	15
8	Vit A (beta carotene) μg	4.66 ± 9.44	28.125	0.0444	850 (μg RE)
9	Phosphorous (milligram)	1075.8 ± 256.4	1584.73	812.4	1000
10	Niacin (milligram)	6.45 ± 1.96	9.437	4.01	17 (mg NE)

### 3.9. Factors Associated With Dietary Practices Among Lactating Mothers

The variables with *p* values < 0.25 in the bivariable analysis were considered candidates for multivariable analysis, and in the multivariable analysis, the variables with *p* values < 0.05 were considered significantly associated with the current dietary practices of lactating mothers who breastfed under 6‐month‐old infants in the Sodo Zuriya district, Wolaita Zone, Ethiopia.

Table 5 shows the socioeconomic and health care–related factors significantly associated with the current dietary practices of lactating mothers, who had access to staple foods available and were counseled about nutrition after giving birth in health institutions, and the frequency of antenatal care visits (Table 5).

**TABLE 5:** Socioeconomic and health care–related factors associated with dietary practices among lactating mothers (*n* = 606).

Variables	Dietary practice		COR (95%CI)	AOR (95% CI)
	Good dietary practice	Poor dietary practice		
Agricultural land				
No agricultural land	41 (61.2%)	26 (38.8%)	3.504 (1.386–8.860)^∗∗^	2.463 (0.894–6.873)
≤ 0.5 ha land	254 (49.8%)	256 (50.2%)	2.205 (0.985–4.935)	1.500 (0.628–3.582)
≥ 0.5 ha land	9 (31.0%)	20 (69.0%)	1	1
Media access				
No media access	192 (53.2%)	169 (46.8%)	1.47 (1.044–2.068)^∗^	1.237 (0.803–1.906)
Listen ≤ 2 times/week	20 (58.8%)	14 (41.2%)	1.848 (0.886–3.854)	1.234 (0.527–2.886)
Listen every day	92 (43.6%)	119 (56.4%)	1	1
Family members				
≤ 5 family members	171 (57.0%)	129 (43.0%)	1	1
6–7 family members	104 (45.8%)	123 (54.2%)	0.638 (0.451–0.902)^∗^	0.663 (0.423–1.041)
> 7 family members	29 (36.7%)	50 (63.3%)	0.438 (0.262–0.730)^∗∗^	0.670 (0.344–1.303)
Place of last delivery				
Home delivery	99 (41.8%)	138 (58.2%)	1.395 (0.747–2.604)	2.31 (0.745–2.781)
Primary health care unit	187 (59.2%)	129 (40.8%)	2.819 (1.530–5.194)^∗∗^	1.292 (0.614–2.178)
Secondary health care unit	18 (34.0%)	35 (66.0%)	1	1
Took iron folic acid				
Not taken at all	32 (34.0%)	62 (66.0%)	1.220 (0.535–2.781)	1.083 (0.414–2.838)
≤ 3 months	261 (54.9%)	214 (45.1%)	2.883 (1.392–5.969)^∗∗^	1.644 (0.725–3.724)
> 3 months	11 (29.7%)	26 (70.3%)	1	1
Staple/food available				
Root tubers and maize	143 (42.9%)	190 (57.1%)	0.465 (0.33–0.655)^∗∗∗^	0.656 (0.436–0.985)^∗^
Cereals and whole grains	17 (42.5%)	23 (57.5%)	0.457 (0.231–0.902)^∗^	0.596 (0.274–1.297)
Both root tubers and cereals and whole grains	144 (61.8%)	89 (38.2%)	1	1
Counseled about nutrition				
Counseled about nutrition	179 (63.3%)	104 (36.7%)	1	1
Not counseled	26 (30.2%)	60 (69.8%)	0.252 (0.150–0.423)^∗∗∗^	0.309 (0.752–0.555)^∗∗∗^
Home delivery $ not asked	99 (41.8%)	138 (58.2%)	0.417 (0.293–0.594)^∗∗∗^	0.632 (0.281–1.418)
Number of ANC				
No ANC visit	12 (22.6%)	41 (77.4%)	0.252 (0.129–0.491)^∗∗∗^	0.405 (0.180–0.909)^∗^
ANC visit ≤ 2 times	18 (41.9%)	25 (58.1%)	0.620 (0.330–1.165)	0.795 (0.393–1.609)
ANC visit 3–4 times or more	274 (53.7%)	236 (46.3%)	1	1

^∗^ *p* < 0.05.

^∗∗^ *p* < 0.01.

^∗∗∗^ *p* < 0.001.

Housing food or the type of staple food available at home increases the likelihood of lactating mothers consuming the recommended additional meal by 34.4% compared with households with only root tubers and households with both root tubers, cereals, and whole grains available [AOR = 0.656, 95% CI [0.436–0.985], *p* value < 0.05].

Counseling about nutrition after giving birth at health institutions increased the recommended additional meal intake among lactating mothers by 69.1% compared with those who did not counsel [AOR = 0.309, 95% CI (0.752–555)], with a *p* value < 0.001.

With respect to antenatal care visits during the last pregnancy, lactating mothers who had no ANC visit during the last pregnancy were 59.5% less likely to have a recommended additional meal than mothers who had ANC visits 3–4 times or more [AOR = 0.405, 95% CI (0.180–0.909)] with a *p* value < 0.05.

The factors of knowledge about food groups being consumed as additional meals and any food group avoided for traditional or cultural reasons were significantly associated variables, as shown in Table 6.

**TABLE 6:** Knowledge and attitudes associated with the dietary practices of lactating mothers.

Variables		Dietary practice		COR (95% CI)	AOR (95% CI)
		Good dietary practice	Poor dietary practice		
Food groups to be taken as additional	Do not knows	2 (15.4%)	11 (84.6%)	0.175 (0.030–0.797)^∗^	0.144 (0.027–0.765)^∗^
	Knows	302 (50.9%)	291 (49.1%)	1	1

Is taking an additional meal good?	Yes	293 (52.2%)	268 (47.8%)	1	1
	No	11 (24.4%)	34 (75.6%)	0.296 (0.147–0.596)^∗∗^	1.061 (0.312–3.607)

Did you think being undernourished due to breastfeeding	Yes	288 (52.8%)	257 (47.2%)	1	1
	No	16 (26.2%)	45 (73.8%)	0.424 (0.265–0.677)^∗∗∗^	1.853 (0.885–3.879)

Did you avoid any food group	Yes	63 (32.0%)	134 (68.0%)	0.328 (0.229–0.469)^∗∗∗^	0.494 (0.321–0.761)^∗∗^
	No	241 (58.9%)	168 (41.1%)	1	1

Are different types of food good?	Yes	291 (52.9%)	259 (47.1%)	1	
	No	13 (23.2%)	43 (76.8%)	0.269 (0.142–0.512)^∗∗∗^	0.553 (0.251–1.217)

Can maternal feeding affect breast milk?	Yes	274 (53.3%)	240 (46.7%)	1	1
	No	30 (32.6%)	62 (67.4%)	0.317 (0.175–0.575)^∗∗∗^	0.537 (0.180–1.608)

^∗^ *p* < 0.05.

^∗∗^ *p* < 0.01.

^∗∗∗^ *p* < 0.001.

Mothers who had no knowledge that food groups would be eaten as additional meals were 85.6% less likely to consume the recommended additional meal than their counterparts were [AOR = 0.144, 95% CI (0.027–0.765)], with a *p* value < 0.05.

For cultural/traditional reasons, mothers who avoided any food group were 50.6% less likely to consume the recommended additional meal during their lactation [AOR = 0.494, 95% CI (0.321–0.761)] than mothers who did not avoid any food group from their meal, with a *p* value < 0.01.

## 4. Discussion

This community‐based study demonstrated that half (49.8%) of lactating mothers in Sodo Zuriya district have poor dietary practices based on additional meal intake. This finding is in line with comparative studies conducted in Raya district, Tigray region, Ethiopia (54.8%) [13].

However, this percentage was lower than those reported in studies conducted in Samre, Raya Woreda, the Tigray region, and Addis Ababa in Ethiopia in 2013 and in Kolkata Hospital, India, in 2017, which were 71.2%, 59.2%, 69.7%, and 59.6%, respectively [9, 13, 27, 28]. This may be due to socioeconomic and sociocultural differences.

The results of the present study also revealed that knowledge about food groups to be taken as additional, avoidance of food groups for cultural/traditional reasons, counseling about nutrition after delivery at health institutions, antenatal care during the previous pregnancy, and the type of staple food available were significantly associated with the dietary practices of lactating mothers.

The results revealed that 72.3% of lactating mothers did not consume a minimum diverse diet, which was higher than the findings of studies conducted in the Wolaita Zone, SNNPR, and Axum town, Tigray region, Ethiopia, which reported that 47.4% and 56.4%, respectively, of lactating mothers had low dietary diversity scores [29, 30]. However, these findings were lower than those of studies conducted in the lowland and highland areas of Raya Woreda, Tigray region, Ethiopia, where 89.9% and 99.6% of lactating mothers, respectively, had low dietary diversity scores [13]. This may be due to variations in socioeconomics, time intervals, and nutritional awareness.

This study revealed the intake of nutrients such as energy (1586.45 ± 374.13 kcal), protein (31.57 ± 10.62 g), calcium (323.1 ± 126.9 mg), and vitamin A or beta carotene (4.66 ± 9.4 µgRE) from lactating mothers. This finding is in line with the findings of a study conducted in the Jimma zone that reported that the macronutrient and energy contents of the diets were below the recommended values [31]. However, this finding is lower than the findings of studies conducted in Tigray, Ethiopia, and the WHO/FAO daily recommendation, except for phosphorus, whose mean value was higher than that of the RDA [9, 23]. This finding is also lower than the findings of studies conducted in Umuahia, Nigeria [32]. This might be due to different sociocultural food crops and their nutrient contents and may also be due to lower economic affordability.

The percentage of ANC visits of four or more times in this study was 44.4%, which is higher than the national DHS report of 32% [33]. This study also revealed that lactating mothers who had mothers who had no ANC visit during their last pregnancy were 59.5% less likely to consume the recommended diet than those who had an ANC visit of 3–4 times or more. This finding is in agreement with the findings of studies conducted in Tigray and Rayitu districts. Ethiopia reported that lactating mothers who had ANC visits equal to or less than 3 times were 2.9 times more likely to be malnourished (BMI < 18.5 kg/m^2^) than those who had visited more than 3 times, and pregnant and lactating mothers who had no ANC were 1.83 times more likely to be malnourished [9, 34]. Another study reported that the prevalence of anemia among pregnant mothers who visited antenatal care frequently was 51% less likely than that among those who had not visited antenatal care [35]. This might be because mothers who attend antenatal care more often during pregnancy may obtain more information from health professionals about their nutritional care or may have better awareness, both in ANC and in feeding practices during pregnancy and lactation.

In contrast, a study conducted in Western Kenya reported that there was no statistically significant difference in dietary diversity between mothers who attended ANC clinics and those who did not attend ANC clinics [36]. This might be due to the low percentage of quality antenatal care or the low level of advice and nutritional education on service delivery.

This study clearly indicates that lactating mothers whose HH produced/available only roots and tubers were 34.4% less likely to practice recommended feeding than those whose HH produced root tubers, cereals, and whole grains. This finding is consistent with the findings of studies by Tigray and Wondo‐Genet, Ethiopia, who reported that non‐maize‐growing mothers were 3 times more likely to be malnourished and that mothers who were not growing vegetables or fruits were 4 times more likely not to have additional meals during pregnancy [9, 37]. This might be because mixed cultivation methods have a positive effect on mothers’ feeding practices.

The lactating mothers who were not counseled about nutrition after delivery in health institutions were 69.1% less likely to consume the recommended diet. This finding agrees with the findings of studies performed in Womberma woreda and Nekemete, Ethiopia, which revealed that women who did not receive nutritional education programs and who had concepts about food were 5 times more likely and 47.9% less likely to be underweight than those who received nutritional education and mothers who had insufficient information about food, respectively [38, 39]. This finding is also consistent with the findings of a study conducted in Aleta Chuko Woreda, South Ethiopia, which reported a significant association of advising or counseling about additional meal intake during antenatal care or pregnancy with improvements in the nutritional status of mothers [40].

Lactating mothers who do not know foods to be consumed as additional meals during lactation were 85.6% less likely to practice recommended diets than those who know about them. Three other studies involving lactating and pregnant mothers in Ethiopia, Eastern Wolega, Addis Ababa, and Bahir Dar town, revealed this association [11, 28, 41], and two countries in Kenya, Turkana County and Nairobi, reported the significant influence of nutritional knowledge on maternal dietary intake and nutritional status [42, 43].

The findings of the study revealed that mothers who avoided any food group for cultural/traditional reasons were 50.6% less likely to consume the recommended diet during their lactating period than their counterparts. This finding is in line with the findings of a study in Wondogenet, southern Ethiopia, that reported that food restriction is significantly associated with the additional meal intake of pregnant mothers [37]. This finding also agrees with the findings of a study conducted in Sudan among pregnant mothers attending antenatal clinics [44]. A study performed in Nigeria revealed the associations between food taboos and the nutritional practices of pregnant mothers [45]. This may be because avoiding any food from their diet had the effect of reducing the alternative feeding of mothers on all available food types and preventing them from consuming meals, even though only culturally/traditionally avoidable foods were available.

### 4.1. Strengths and Limitations of This Study

#### 4.1.1. Strength

A larger sample size was used than in previous studies.

Multiple tools have been used to assess the current dietary practices of lactating mothers.

#### 4.1.2. Limitations

The dependent variable was dichotomized on the basis of the interview response and considered only recommended additional meal intake.

This research did not measure the amount of extra energy consumed.

The dietary data were collected in the same season.

## 5. Conclusion and Recommendation

### 5.1. Conclusion

Nearly half of lactating mothers had poor dietary practices according to national or WHO recommendations. Counseling about nutrition after delivery, frequency of antenatal care visits, avoidance of food groups during lactation, knowledge of food groups to be eaten as additional meals, and staple food types available or produced were significantly associated with the dietary practices of lactating mothers.

### 5.2. Recommendation

A. To district health offices and health care providers:
 Strengthen routine postnatal nutritional counseling.
 Strengthening ANC coverage and integrate lactation‐specific nutritional education into routine maternal service.
B. To Agricultural Office
 Promote mixed crop cultivation practice at the household level, and encourage families to diversify foods by including legumes, green vegetables, and cereals.
C. To public health programs
 Design targeted community SBCC (Social and Behavioral Change Communication) campaigns involving elders, community, and religious leaders to dispel traditional food taboos and promote nutritious local food consumption during lactation.

**Nomenclature**
AOR: Adjusted odds ratio
BMI: Body mass index
COR: Crude odds ratio
FAO: Food and Agriculture Organizations
UNICEF: United Nations International Children’s Fund
WHO: World Health Organization

## Author Contributions

Conceptualization, H.E.J. and M.A.A.; data curation, H.E.J., M.A.A., and T.T.; formal analysis, H.E.J., M.A.A., and T.T.; funding acquisition, M.A.A.; investigation, H.E.J., M.A.A., and T.T.; methodology, H.E.J., M.A.A., and T.T.; project administration, M.A.A.; resources, H.E.J. and M.A.A.; software, H.E.J. and M.A.A.; supervision, M.A.A. and T.T.; validation, H.E.J. and M.A.A.; visualization, M.A.A. and T.T.; writing–original draft, H.E.J. and M.A.A.; writing–review and editing, H.E.J., M.A.A., and T.T.

## Funding

No funding was obtained.

## Ethics Statement

Official ethical clearance was obtained from Wolaita Sodo University’s ethical review board, and informed verbal consent was obtained from the study participants.

## Consent

Informed consent was obtained from all subjects involved in the study.

## Conflicts of Interest

The authors declare no conflicts of interest.

## Data Availability

The data presented in this study are available on request to the corresponding author.

## References

[1] Griffin P. B. , Maternal Nutrition During Lactation by Paula Benedict Griffin, 2010.

[2] Nikolaos K. M. C. , Sci M. M. , Meropi K. , Evangelia M. et al., Clinical Nutrition in Practice, 2010, Blackwell Publishing Ltd.

[3] Gwavuya S. , Murendo C. , Wekwete N. , Takavarasha F. , and Madzingira N. , Maternal Iron and Vitamin A Supplementation and the Nutritional Status of Children, ICF International Rockville. (August 2014) 10, Maryland.

[4] Ransom I. E. E. K. , Nutrition of Women and Adolescent Girls: Why it Matters? Population Reference Bureau, 2003.

[5] Valeggia C. R. and Ellison P. T. , Impact of Breastfeeding on Anthropometric Changes in Peri-Urban Toba Women (Argentina), American Journal of Human Biology. (2003) 15, no. 5, 717–724, 10.1002/ajhb.10202. 12953184

[6] Black R. E. , Allen L. H. , Bhutta Z. a. et al., Maternal and Child Undernutrition: Global and Regional Exposures and Health Consequences, 2008. 10.1016/S0140-6736(07)61690-0 18207566

[7] Zerfu T. A. , Dietary Practices of Pregnant Women and Its Associations With Maternal and Perinatal Outcomes in Rural Central Ethiopia, Center for Food Science and Nutrition College of Natural Sciences Addis Ababa University. (2016) .

[8] Centers E. , Wollega E. , Temesgen D. , Hundera F. G. H. , and Tesso D. , Nutritional Knowledge and Determinant Factors Among Lactating Mothers in Nekemte Referral Hospital and Health, Food Science and Quality Management. (2015) 38.

[9] Haileslassie K. , Mulugeta A. , and Girma M. , Feeding Practices, Nutritional Status and Associated Factors of Lactating Women in Samre Woreda, South Eastern Zone of Tigray, Ethiopia, Nutrition Journal. (2013) 12, no. 1, 10.1186/1475-2891-12-28. PMC3599359 23452646

[10] Weldehaweria , Misgina K. H. , Weldu M. G. et al., Dietary Diversity and Related Factors Among Lactating Women Visiting Public Health Facilities in Aksum Town, Tigray, Northern Ethiopia, BMC Nutrition. (2016) 2, no. 1, 10.1186/s40795-016-0077-3.

[11] Temesgen Desisa Hundera1 , Nutritional Knowledge and Determinant Factors Among Lactating Mothers in Nekemte Referral Hospital and Health Centers, East Wollega, Ethiopia, 2015.

[12] National Academy of Sciences Nutrition During Lactation , Food and Nutrition Board Institute of Medicine National Academy of Sciences, 2013.

[13] Sitotaw , Hailesslasie K. , and Adama Y. , Comparison of Nutritional Status and Associated Factors of Lactating Women Between Lowland and Highland Communities of District Raya, Alamata, Southern Tigiray, Ethiopia, BMC Nutrition.(2017) 3, no. 1, 10.1186/s40795-017-0179-6. PMC7050921 32153841

[14] Kennedy TbaMD G. , Guidelines for Measuring Household and Individual Dietary Diversity. Nutrition and Consumer Protection Division, Food and Agriculture Organization of the United Nations. (2013) .

[15] Agency C. S. , Addis Ababa E. , International I. , and Calverton M. , Ethiopia Demographic and Health Survey, 2011.

[16] MsfncwPGPDFND Y. F. M. R. D. , Guidelines for Assessing Nutrition-Related Knowledge, Attitudes and Practices, Food and Agriculture Organization of the United Nations. (2014) .

[17] et.al DpRm , Research Methodology, 2013, Himalaya Publishing House.

[18] Health FMo , National Nutrition Strategy, 2016.

[19] Usaid F. A. , Minimum Dietary Diversity for Women, Monitoring and Evaluation. (2016) 3, FANTA.

[20] EHNRI , Food Composition Table for Use in Ethiopia, 1999.

[21] Rosa M. , Ortega1 CP-RaAML-S. Dietary Assessment Methods: Dietary Records, Nutricion Hospitalaria. (2015) 31, 38–45. 25719769 10.3305/nh.2015.31.sup3.8749

[22] Ferro-Luzzi A. , Individual Food Intake Survey Methods, National Institute for Food and Nutrition Research Rome, Italy.

[23] WHO/FAO , Human Vitamin and Mineral Requirements, A Report of Joint FAO/WHO Expert Consultation Committee: 2002. (2002) WHO.

[24] Linkages (USAID) , Breastfeeding and Maternal Nutrition Frequently Asked Questions (FAQ), AED. (July 2003) 4.

[25] project l ENA Counselor’s guide , Key Behaviors for Optimal Breast Feeding, Complementary Feeding and Maternal Nutrition at Critical Stages in the Life Cycle of Women and Children Draft for Discussion, 2008, http://motherchildnutritionorg/nutrition//mcn-ena-key-messages-bookl.

[26] WHO , Healthy Eating during Pregnancy and Breastfeeding, Nutrition and Food Security. (2001) WHO.

[27] Mallik Dr Sarmila and Majumdar Sukanta , A Study on Nutritional Status of Lactating Mothers Attending the Immunization Clinic of a Medical College Hospital of Kolkata, West Bengal, IOSR Journal of Dental and Medical Science. (july 2017) 16, 30–34.

[28] Zelalem , Endeshaw M. , Ayenew M. , Shiferaw S. , and Yirgu R. , Effect of Nutrition Education on Pregnancy Specific Nutrition Knowledge and Healthy Dietary Practice Among Pregnant Women in Addis Ababa, Clinics in Mother and Child Health. (2017) 14, no. 3, 10.4172/2090-7214.1000265.

[29] Weldehaweria N. B. , Dietary Diversity and Related Factors Among Lactating Women Visiting Public Health Facilities in Aksum Town, Tigray, Northern Ethiopia. (2017) .

[30] Asefa6 BWJAHGASEDKT , Chronic Energy Deficiency and Associated Factors Among Lactating Mothers (15–49 Years Old) in Offa Woreda, Wolayita Zone, SNNPRs, Ethiopia, World Scientific Research. (2018) 5.

[31] Sirawdink F. and TbaOH F. , Fighting Hidden Hunger: Diversity, Composition and Nutrient Adequacy of Diets of Lactating Mothers in Jimma Zone, Southwest Ethiopia, 2016, BOKU Vienna.

[32] Ukegbu P. et al., A Study of the Nutritional Status and Dietary Intake of Lactating Women in Umuahia, Nigeria, American Journal of Health Research. (February 2014) 2, no. 1.

[33] Central Statistical Agency Addis Ababa ETDPIR , Ethiopia Demographic and Health Survey, Octover. (2016) Maryland, USA.

[34] al Ge , Taddese Z. , Legesse T. , and Letebo M. , Determinants of Malnutrition Amongpregnant and Lactating Women Under Humanitarian Setting in Ethiopia, BMC Nutrition. (2018) 4, no. 1, 10.1186/s40795-018-0222-2. PMC7050776 32153875

[35] Umeta2 TAa , Reproductive and Obstetric Factors are Key Predictors of Maternal Anemia During Pregnancy in Ethiopia: Evidence From Demographic and Health Survey, 2011, Hindawi Publishing Corporation. 10.1155/2015/649815 PMC4568321 26417454

[36] Cole D. C. , Ouédraogo H. Z. , Sindi Kirimi et al., Health and Nutrition Knowledge, Attitudes and Practices of Pregnant Women Attending and Not-Attending ANC Clinics in Western Kenya: A Cross-Sectional Analysis, 2013. 10.1186/1471-2393-13-146 PMC3716969 23845074

[37] K D. , Dietary Patterns and Their Associations With Energy, Nutrient Intake and Socioeconomic Factors in Rural Lactating Mothers in Tibet, Wondo Genet. Journal of Pharanceutical and Scientific Innovation. (2015) . 10.6133/apjcn.012016.13 28429910

[38] Sileshi Berihun GMKaMT , Factors Associated With Underweight Among Lactating Women in Womberma Woreda, Northwest Ethiopia; a Crosssectional Study, BMC Nutrition. (2017) . 10.1186/s40795-017-0165-z PMC7050864 32153826

[39] Temesgen Desisa Hundera D. W. , Fekadu Gemede H. , and Kenie D. N. , Nutritional Status and Associated Factors Among Lactating Mothers in Nekemte Referral Hospital and Health Centers, Ethiopia, International Journal of Nutrition and Food Sciences. (2015) 4, 216–222.

[40] Sonko A. , Assessment of Dietary Practice and Anthropometric Status of Pregnant Women in Aleta Chuko Woreda Southern Nations, Nationalities and People’s Region/SNNPR/, Ethiopia: Descriptive Cross-Sectional Study, Journal of Epidemiology and Public Health Reviews. (January 2016) 10.

[41] Nana A. , Dietary Practices and Associated Factors During Pregnancy in Northwestern Ethiopia, BMC Pregnancy and Childbirth. (2018) . 10.1186/s12884-018-1822-1 PMC5970492 29801471

[42] Ongosi A. N. , Nutrient Intake and Nutrition Knowledge of Lactating Women (0–6 Months Postpartum) in a Low Socio-Economic Area in Nairobi, Kenya, 2010.

[43] TmaM M. , Determinants of Maternal and Child Malnutrition Among Low Income Households in Turkana County, Kenya, FASEB. (2017) .

[44] kheiri S. A. K. A. , Mustafa L. S. , Shaaeldin M. A. , and Alsammani M. A. , Superstitious Food Beliefs and Traditional Customs Among Ladies Attending the Antenatal Clinic at Omdurman Maternity Hospital (OMH), Omdurman, Sudan, Annals of Medical and Health Sciences Research. (2017) 7, 218–221.

[45] Uchenna E.1 C. D. I. O. , Food Taboos and Myths in South Eastern Nigeria: The Belief and Practice of Mothers, Journal of Ethnobiology and Ethnomedicine. (2016) . 10.1186/s13002-016-0079-x PMC4729178 26818243

